# Supplementary material for: Expansion quantization network: A micro-emotion detection and annotation framework
Source: PLoS One. 2025 Nov 13;20(11):e0333930. doi: 10.1371/journal.pone.0333930 (PMC12614796; doi:10.1371/journal.pone.0333930)
Supplement: S1 File — (PDF) [file pone.0333930.s001.pdf]

Pseudocode for the Python Implementation of the EQN Framework (Using the BERT Model as an Example)

```
import torch
from transformers import BertTokenizer, BertForSequenceClassification,
AdamW
from sklearn.model_selection import train_test_split
from imblearn.over_sampling import SMOTE
from imblearn.under_sampling import RandomUnderSampler
import numpy as np


# Step 1: Data Preprocessing and Feature Extraction


def preprocess_data(texts, labels, tokenizer):
    encodings = tokenizer(texts, truncation=True, padding=True,
max_length=512)
    return encodings, labels


# Step 1.1: Addressing the Class Imbalance Issue

def handle_imbalance(X, y, method="oversampling"):
    """

    Use oversampling or undersampling techniques to address the class
imbalance issue.

    method="oversampling"
    method="undersampling"
    """
    if method == "oversampling":
        sample = oversampling()
        X_res, y_res = sample.fit_resample(X, y)
    elif method == "undersampling":
        sample = undersampling ()
        X_res, y_res = sample.fit_resample(X, y)
    else:
        raise ValueError("Invalid method. Choose 'oversampling' or
'undersampling'.")

    return X_res, y_res
```

```
# Step 2: Full Label Initialization

def initialize_full_labels(labels, max_value=10.0, min_value=0.0):
    full_labels = []
    for label in labels:
        initialized_label = [max_value if l == 10 else min_value for l
in label]  # 10 represents labeled, and 0 represents unlabeled.
full_labels.append(initialized_label)
    return full_labels


# Step 3: Input the BERT model for training.

def train_bert_model(train_encodings, train_labels, tokenizer):
    model =
BertForSequenceClassification.from_pretrained('bert-base-uncased',
num_labels=len(train_labels[0]))
    optimizer = AdamW(model.parameters(), lr=1e-5)


    # Convert the training data into tensors.

    train_encodings = torch.tensor(train_encodings)
    train_labels = torch.tensor(train_labels)

    # Start training
    model.train()
    for epoch in range(n):  # Iterate for n epochs.
        optimizer.zero_grad()
        outputs = model(train_encodings, labels=train_labels)
        loss = outputs.loss
        loss.backward()
        optimizer.step()
        print(f"Epoch {epoch + 1} Loss: {loss.item()}")

    return model

# Step 4: Fully connected layer output and linear activation.
def apply_linear_activation(model, inputs):
    outputs = model(inputs)   # Obtain the BERT output.
    last_hidden_state = outputs.last_hidden_state

    logits = last_hidden_state.mean(dim=1)  # Simple example: Average
pooling.
```

```
    return logits


# Step 5: Label regression.

def label_regression(model, train_labels, predicted_labels,
min_value=0.0, max_value=10.0):
    updated_labels = []
    for i, label in enumerate(train_labels):
        updated_label = [predicted_labels[i][j] if label[j] == min_value
else max_value for j in range(len(label))]
        updated_labels.append(updated_label)
    return updated_labels

# Step 6: Retrain the training set after regression.
def retrain_with_regression(model, train_encodings, updated_labels):
    model = train_bert_model(train_encodings, updated_labels,
tokenizer)
    return model


# Step 7: Use the optimal model for sentiment classification prediction.

def predict_sentiment(model, test_texts, tokenizer):
    test_encodings, _ = preprocess_data(test_texts, None, tokenizer)
    test_encodings = torch.tensor(test_encodings)
    model.eval()
    with torch.no_grad():
        outputs = model(test_encodings)
    logits = outputs.logits
    return logits

# Example
texts = ["I love this product!", "This is the worst movie ever."]
labels = [[1, 0, 0], [0, 1, 0]]  # Assume there are three labels.
tokenizer = BertTokenizer.from_pretrained('bert-base-uncased')


# Handling class imbalance

X_resampled, y_resampled = handle_imbalance(texts, labels,
method="oversampling")


# Data preprocessing
```

```
encodings, full_labels = preprocess_data(X_resampled, y_resampled,
tokenizer)


# Full label initialization


initialized_labels = initialize_full_labels(full_labels)


# Train the model


trained_model_1 = train_bert_model(encodings, initialized_labels,
tokenizer)


# Obtain the predicted labels after training.

predicted_labels = np.random.rand(len(full_labels),
len(full_labels[0]))
# Label regression
regressed_labels = label_regression(trained_model_1,
initialized_labels, predicted_labels)

# Retrain
trained_model_2 = retrain_with_regression(trained_model_1, encodings,
regressed_labels)

# Sentiment prediction and labeling.
test_texts = ["I hate this place!"]
predictions = predict_sentiment(trained_model_2, test_texts, tokenizer)
print(predictions)
```
