# Supplementary material for: Expansion quantization network: A micro-emotion detection and annotation framework
Source: PLoS One. 2025 Nov 13;20(11):e0333930. doi: 10.1371/journal.pone.0333930 (PMC12614796; doi:10.1371/journal.pone.0333930)
Supplement: Attachment — Submitted filename: Response_to_Reviewers_auresp_2.docx [file pone.0333930.s004.docx]

**Dear editor and reviewers:**

Thank you for taking the time to review my manuscript and for providing valuable feedback.
Below is a detailed response to reviewer’s comments, outlining the modifications made and the corresponding explanations. I hope these revisions will further enhance the quality of the paper and meet the journal's requirements.

**Reviewer #3: Some small things should still be addressed/discussed:
First,** the EQN pipeline-full-label initialization, regression, and training-set annotation-has so many pieces that other researchers may struggle to set it up exactly as written.

**Response:** We appreciate the reviewer’s insightful feedback. Indeed, the original description of the EQN process was somewhat verbose and may have caused confusion. Now, we have revised the corresponding section of the manuscript (see Lines 266–289 in the revised version). The updated description is now clearer, more structured, and more accessible for readers to follow.
The specific revisions are as follows:
The EQN framework consists of a two-stage training pipeline for enhancing emotion classification through full-label regression. Its operation is straightforward and can be summarized as follows:

1. Data Preparation: Each training sample with a single emotion label is transformed into a multi-dimensional one-hot vector representing the full emotion label space (e.g., for 28 emotions in GoEmotions).
2. Stage 1 – Full Label Initialization & Model A Training:

A base classification model (Model A) is trained on the initialized dataset using standard loss functions (e.g., MSE loss for regression).

Model A learns to map input texts to emotion label vectors.

3. Soft Label Generation:

Model A is used to predict probability distributions over the entire label space for each training sample.

These predicted probabilities serve as soft labels for all emotions not originally annotated (while preserving the original label as 1.0).

4. Stage 2 – Model B Training with Refined Labels:

A second model (Model B) is trained on the soft-labeled dataset to learn a more robust and generalized representation of emotional features.

5. Inference:

Model B can be used to classify emotions of new texts or assign multi-dimensional emotional scores for downstream tasks.

Note: Steps 1–3 are referred to as the core EQN module (CoEQN), which is also used as a standalone component in Section 4 for ablation experiments.
A full Python-style pseudocode is provided in the Supplementary Material to ensure reproducibility.
We believe this clearer description, along with the accompanying pseudocode, ensures that researchers can implement the EQN framework without difficulty.

**Second**, the validation sets- GoEmotions, Thealth, Gemotions-cover few emotion classes, leaving some feelings seriously underrepresented. That gap weakens claims about robustness.

**Response**: Thank you for your valuable comment. We fully understand your concern regarding the limited coverage of emotion categories in the validation datasets.Our study has deliberately incorporated a diverse range of datasets that span various levels of emotional granularity (3, 6, 7, and 28 classes), ensuring both breadth and representativeness in our robustness evaluation of the EQN framework. These datasets include a wide range of emotional domains, from basic emotions to fine-grained states, and cover multiple real-world application areas. Specifically, we used the following datasets:

- **7health dataset**: Related to mental health, containing 51,074 entries labeled with seven categories—anxiety, bipolar disorder, depression, normal, personality disorder (PD), stress, and suicidal ideation.
- **6emotions dataset**: An English corpus annotated with six basic emotions—sadness, joy, love, anger, fear, and surprise.
- **3TFN dataset**: Financial domain tweets annotated for sentiment polarity.
- **3TSA dataset**: Approximately 163,000 tweets labeled with three sentiment categories—positive, neutral, and negative.
- **GoEmotions dataset**: Released by Google, featuring 28 fine-grained emotion labels. It is one of the most detailed and comprehensive publicly available emotion datasets.

By combining these datasets, our study covers a wide range of emotional contexts—including human affective states, mental health, financial sentiment, and social media emotion analysis—with both coarse- and fine-grained labels. Therefore, we believe the robustness evaluation of EQN is well-grounded and broadly representative.

**Third,** because BERT carries most of the load (model dependency), I wonder whether the approach transfers to models like ANN, CNN, LSTM, or TextCNN that the team did test. BERTs dominance still hints at a narrow sweet spot.

**Response:** We appreciate the reviewer’s thoughtful concern regarding potential model dependency in our approach. Our proposed framework **EQN is designed to be model-agnostic**, and we thoroughly tested it across five diverse classification architectures: ANN, CNN, TextCNN, LSTM, and BERT.

To directly address the concern of model transferability, we present in Table 3 a comprehensive comparison of three classification strategies—conventional single-label (S), multi-label (M), and our EQN full-label mapping approach (EQN)—evaluated on five datasets. The results demonstrate that EQN consistently outperforms both S and M baselines across all tested models, including those with no pretrained components (ANN, CNN, TextCNN, LSTM, BERT).

**Table 3.Testing results of single-label, multi-label, and full-label mapping classification models.**

| model/dataset |  | 7health | 6emotions | **3TFN** | **3TSA** | 28GoEmotions |
| --- | --- | --- | --- | --- | --- | --- |
| ANN | S | 0.3202 | 0.3358 | 0.6557 | 0.5199 |  |
|  | M |  |  |  |  | 0.1611 |
|  | EQN | 0.374 | 0.3551 | 0.6482 | 0.5254 | 0.1877 |
| CNN | S | 0.6658 | 0.6778 | 0.7353 | 0.8166 |  |
|  | M |  |  |  |  | 0.3892 |
|  | EQN | 0.6836 | 0.7113 | 0.7592 | 0.8161 | 0.4076 |
| TextCNN | S | 0.6624 | 0.7603 | 0.7575 | 0.7357 |  |
|  | M |  |  |  |  | 0.3889 |
|  | EQN | 0.6672 | 0.7869 | 0.7717 | 0.7448 | 0.4205 |
| LSTMS | S | 0.7068 | 0.9318 | 0.8099 | 0.93 |  |
|  | M |  |  |  |  | 0.4545 |
|  | EQN | 0.7295 | 0.9352 | 0.8073 | 0.9283 | 0.4658 |
| BERT | S | 0.7855 | 0.9328 | 0.8378 | 0.9405 |  |
|  | M |  |  |  |  | 0.4665 |
|  | EQN | 0.8034 | 0.935 | 0.8458 | 0.9413 | 0.4849 |

**Table notation**: single-label is denoted as S, multi-label as M, and EQN full label mapping as EQN.

An illustrative example is provided based on the experimental results in Table 3.:

- On the **GoEmotions** dataset, EQN improves performance over multi-label classification by:

+0.0266 on **CNN** (from 0.3892 to 0.4076),
+0.0316 on **TextCNN** (from 0.3889 to 0.4205),
+0.0113 on **LSTM** (from 0.4545 to 0.4658),
+0.0184 on **BERT** (from 0.4665 to 0.4849).

- On **6emotions**, EQN outperforms single-label classification by:

+0.0193 on **ANN**,
+0.0335 on **CNN**,
+0.0266 on **TextCNN**,
+0.0034 on **LSTM**,
+0.0022 on **BERT**.

The analysis of the above data indicates that, from the perspective of relative performance improvement, BERT does not have an advantage over other models after applying the EQN framework. The reason for choosing BERT is that it generally achieves better performance than other models in natural language processing tasks. This supports our claim that EQN is broadly applicable and not confined to a narrow sweet spot.

**Last**, despite the title, the paper says little about what micro-emotions are or gives concrete examples. This ambiguity may puzzle readers and, in turn, shrink the papers impact.

**Response:**
Thank you for your insightful comment. In the revised manuscript, we have added a detailed explanation and concrete examples of *micro-emotions* in the Introduction section (lines 107–117) to improve clarity and reduce potential reader confusion.

The supplementary content added in the revised manuscript is as follows:

“In the context of natural language, micro-emotions refer to fleeting, low-intensity, and often subconscious emotional states expressed subtly through text. These emotions are typically harder to detect than macro-emotions such as joy or anger, as they are conveyed through nuanced wording, implicit sentiment, or slight linguistic variations. For instance, a sentence like "That's exactly the kind of brilliant nonsense I expected" may carry sarcasm mixed with disappointment and helplessness—emotional shades that would be missed by coarse labeling systems. Micro-emotions provide a deeper view into the speaker’s internal psychological state, making them highly valuable in fields such as sentiment analysis, mental health screening, and user experience optimization. Our study considers micro-emotions as integral components of fine-grained emotion modeling and aims to capture them systematically through quantification and annotation.”
